# The relationship between lipid risk score and new-onset hypertension in a prospective cohort study

**DOI:** 10.3389/fendo.2022.916951

**Published:** 2022-09-28

**Authors:** Hankun Xie, Qian Zhuang, Jialing Mu, Junxiang Sun, Pengfei Wei, Xianghai Zhao, Yanchun Chen, Jiayi Dong, Changying Chen, Lai Wei, Yunjie Yin, Song Yang, Chong Shen

**Affiliations:** ^1^ Department of Epidemiology, School of Public Health, Nanjing Medical University, Nanjing, China; ^2^ Department of Cardiology, Affiliated Yixing People’s Hospital of Jiangsu University, People’s Hospital of Yixing City, Yixing, China

**Keywords:** dyslipidemia, hypertension, risk score, lipid indices, cohort study

## Abstract

**Background:**

Dyslipidemia and hypertension are both important risk factors for atherosclerotic cardiovascular diseases. However, the relationship between dyslipidemia and incident hypertension remains to be elucidated comprehensively. The main purpose of this study was to construct the lipid risk score to explore the risk prediction effect of integrated lipid indices on new-onset hypertension.

**Methods:**

This prospective cohort study with 2116 non-hypertensive subjects was conducted from 2009 to 2020. New hypertension events during the follow-up period were recorded and verified. The lipid risk score was calculated by summing coded total cholesterol, triglyceride, low-density lipoprotein cholesterol, and high-density lipoprotein cholesterol weighted with corresponding effect sizes. Cox regression analysis was used to estimate the association between the lipid risk score or lipid indices and incident hypertension in the subgroup of age (< 55 and≥ 55 years at baseline).

**Results:**

After a median of 10.75-year follow-up, 637 incident hypertension cases were identified. The restricted cubic spline showed that the lipid risk score had a positive linear correlation with hypertension (P< 0.001). Among people< 55 years, with every increase of 0.94 in lipid risk score, the risk of hypertension increased by 37% (adjusted HR [95%CI]: 1.369 [1.164-1.610]). This association was not modified by overweight or obesity.

**Conclusions:**

The integrated lipid risk score, independent of traditional risk factors, has a significantly predictive effect on hypertension in people younger than 55 years. This finding may aid in identifying high-risk individuals for hypertension, as well as facilitating early intervention and management to reduce adverse cardiovascular events. Comprehensive lipid management should be attached importance in the prevention and control of hypertension.

## Introduction

Hypertension is a serious public health issue that serves as a primary risk factor for cardiovascular disease and a major cause of premature death worldwide (1, 2). An estimated 1.28 billion people aged 30-79 years globally have hypertension by 2019 (3). In China, more than 23.2% of adults suffer from hypertension, but only 15.3% of hypertensive patients achieved blood pressure control (4). The direct socioeconomic burden caused by hypertension remains huge due to its high prevalence, low awareness, and insufficient controls (5). Under this circumstance, the latent burden of cardiovascular disease brought about by hypertension is worsening in China. Currently, there is a growing potential for primary prevention and early detection to prevent the development of hypertension and increase in cardiovascular risk (6, 7). Hence, investigations aimed at identifying risk factors or developing prediction models for hypertension are imperative, which will help ensure early intervention and management to reduce adverse cardiovascular events related to hypertension.

Dyslipidemia, characterized by abnormalities of serum cholesterol or triglyceride levels, is also an established risk factor for atherosclerotic cardiovascular diseases (8). The coexistence of dyslipidemia and hypertension frequently occurs in clinical practice (9–13). Accumulating evidence has supported the pathophysiological interplay between hypertension and dyslipidemia, which involves oxidative stress, proinflammatory activities, stimulation of the renin-angiotensin-aldosterone system (RAAS), and endothelium dysfunction (9, 10, 14). Furthermore, a number of prospective epidemiological studies consistently suggest a causal relationship between abnormality in blood lipid levels and the risk of future incident hypertension (15–22). Some researchers found that elevated levels of total cholesterol (TC), triglyceride (TG), low-density lipoprotein cholesterol (LDL-C), or non-high-density lipoprotein cholesterol (non-HDL-C), and a lower level of high-density lipoprotein cholesterol (HDL-C) increase the risk of developing hypertension (20–22). However, most studies focused on the relationship between a single lipid indicator and hypertension rather than the association of combined lipid parameters with hypertension. Research on the combined effects of multiple lipid indices on hypertension is limited, which necessitates further exploration.

Developing risk prediction models for hypertension is an important approach to identifying high-risk individuals and conducting targeted interventions. To date, plenty of prediction models have been constructed and validated for various populations. The variables included in these risk models mainly involve traditional risk factors or genetic factors, yet few models incorporate lipid indices (23). Thus, research regarding lipid profiles and the risk of hypertension might be conducive to refining the prediction model and providing a more comprehensive risk assessment for hypertension.

Therefore, the primary objective of this study was to evaluate the association between abnormalities of multiple lipid indices and new-onset hypertension in a prospective cohort, and construct the lipid risk score to explore the combined risk-prediction effect of integrated lipid profiles on hypertension.

## Materials and methods

### Research design and study population

This prospective cohort research commenced from May 2009 in China, which recruited a total of 4128 subjects by a cluster sampling approach from 6 villages in Yixing City, Jiangsu Province. Eligible participants included at baseline were community residents aged 24 to 96 years old. 2116 baseline subjects free of hypertension (systolic blood pressure:< 140 mmHg; diastolic blood pressure:< 90 mmHg) were followed up until July 27, 2020 for hypertension status.

The study has been approved by the Ethics Committee of Nanjing Medical University in Jiangsu Province of China, and all participants signed the written informed consent before entering the study cohort. Data and information of every subject in the current study were de-identified and kept confidential.

### Baseline survey and data collection

The baseline investigation for all eligible participants was administered by well-trained and qualified researchers at the time of enrollment. Each subject completed a validated questionnaire with demographic information, smoking/drinking status, family history, and medical history of chronic diseases including hypertension. Smokers were defined as individuals having more than 20 cigarettes/week for at least 3 months per year. Drinkers in this study referred to individuals with current or past alcohol consumption more than two times per week lasting at least 6 months a year. All subjects received physical examinations conducted by professional doctors or nurses for basic physiological information. Height, weight, and blood pressure were measured by calibrated instruments. Body mass index (BMI) was calculated by the ratio of weight in kilograms to the square of height in meters, with< 24 kg/m^2^ as normal or underweight, 24-27.9 kg/m^2^ as overweight, and ≥ 28 kg/m^2^ as obese. Blood pressure measurements for all participants were conducted in the morning (7:00-10:00 am) after at least 8-hour self-administered fasting. Mercury sphygmomanometers were used on the right arm to read the blood pressure value at least three times after a 5-minute rest in the sitting position. The final blood pressure was the average value of three measurements. Individuals with fasting plasma glucose (FPG) ≥ 7.0 mmol/L, or self-reported diabetes diagnosis, or currently receiving antidiabetic medication were recorded as diabetes cases. After data collection, we used EpiData 3.0 software for double entry and consistency check to ensure the authenticity and accuracy of the entered information.

### Lipid indices measurement and categorization

Overnight fasting blood samples (10mL) were collected from all participants and transported to the hospital within 24 hours for laboratory tests. TC, TG, HDL-C, LDL-C, and FPG were measured by an automatic biochemical analyzer. Non-HDL-C was calculated as the difference value of TC and HDL-C.

Blood lipid levels were categorized in the light of Chinese Guidelines for the Management of Dyslipidemia in Adults (24). We classified the level of TC, TG, LDL-C, and non-HDL-C as normal, marginal increase, and increase according to the recommended cut-off values for the Chinese population (200 mg/dl and 240 mg/dl for TC; 150 mg/dl and 200 mg/dl for TG; 130 mg/dl and 160 mg/dl for LDL-C; 160 mg/dl and 190 mg/dl for non-HDL-C). HDL-C level was classified as normal, low, and high by the cut-off points of 40 mg/dl and 60 mg/dl. Dyslipidemia was defined as abnormal changes in any of the four lipid indices (TC ≥ 240 mg/dl, or TG ≥ 200 mg/dl, or LDL-C ≥ 160 mg/dl, or HDL-C< 40 mg/dl), or self-reported diagnosis of dyslipidemia, or currently taking lipid-lowering drugs.

### Follow up and outcome detection

The first field follow-up investigation for baseline participants began from May to October in 2014 with the same approach and content as the baseline survey. The follow-up proceeded until July 27, 2020, with annual regular monitoring for hypertension events. Individuals with an average systolic blood pressure (SBP) ≥ 140 mmHg or diastolic blood pressure (DBP) ≥ 90 mmHg were diagnosed with hypertension during the follow-up. The incidence of hypertension was recorded strictly according to the local management system of disease and death of the Center for Disease Control and Prevention (CDC) or the regular registration in community health centers. All monitored hypertension events were further verified by the end-point committee.

### Lipid risk score calculation

We constructed the lipid risk score in the population under 55 years at baseline by integrating four lipid indices (TC, TG, LDL-C, and non-HDL-C) which were significantly associated with the risk of hypertension. Each individual aged< 55 years was assigned a score based on the levels of selected four lipid indices. Each of the four lipid indices was assigned corresponding code. Normal lipid levels were coded as 0, and marginal increase or increase of lipid indices was coded as 1. Due to the unequal effects of different lipid indices and different lipid levels on the risk of hypertension, we introduced the weight or effect size for each category of four lipid indices into the calculation of the lipid risk score. Adjusted Cox regression analyses between lipid indices and hypertension incidence were conducted to determine the effect of different lipid levels on hypertension risk by the estimate of β coefficient. β estimates for every category of lipid indices from multivariable Cox regression analyses are listed in **Table 3**. Based on results of the association between TG and hypertension in this study and the heterogeneity test for TG groups, the weight for TG was the averaged β estimate generated by combining the categories of marginal increase (150-200 mg/dl) and increase (>200 mg/dl). Then, the lipid risk score for each individual was calculated by summing the multiplication value of the code (0 or 1) and corresponding weights (β estimates) of four lipid indices.

### Statistical analysis

We first evaluated the distribution of baseline characteristics of the study population by whether the participant had dyslipidemia. Continuous variables were presented as medians and interquartile range for the non-normality of distribution, and the Mann-Whitney U test was performed to examine the differences between the two groups. Categorical variables were presented as frequencies and proportions, and differences between two groups were compared using the Chi-square (*χ* ^2^.)test. Incidence density was calculated by the ratio of the number of incident hypertension cases to person-years. Bivariate correlations between lipid indices and hypertension were also analyzed by *χ* ^2^ test. Cox proportional hazard regression was applied to calculate the hazard ratio (HR) with 95% confidence interval (CI), so as to test whether significant associations exist between blood lipid levels and the risk of hypertension in two age groups (< 55 and ≥ 55 years at baseline) after adjusting for age, gender, smoking, drinking, hypertension family history, baseline diabetes.

Restricted cubic spline regression with five knots was used to model the association curve of lipid risk score with hypertension risk, as well as test for linearity or non-linearity. The lipid risk score was then classified with equal intervals to generate four risk groups for hypertension. Cox regression analyses were also performed to assess the association between lipid risk score and incident hypertension. Concordance statistics (C-statistics) for Cox regression were computed to evaluate the improvement in discrimination after adding the lipid risk score to the traditional prediction model for hypertension. The likelihood ratio test was conducted to compare the goodness of fit of the two models.

Statistical analyses were carried out using SAS 9.4 software (SAS Institute Inc., Cary, NC, USA) and R software 3.5.0 (R Foundation for Statistical Computing, Vienna, Austria). Analysis results were considered statistically significant at the two-sided 5% level.

## Results

### Population characteristics and hypertension incidence by dyslipidemia

As reported in **Table 1**, among the study population of 2116 individuals, which consisted of 853 males (40.3%) and 1263 females (59.7%), 856 subjects were younger than 55 years at baseline, and 408 had a family history of hypertension (19.3%). The median BMI of the total population was 23.42 kg/m^2^. 31.9% of the participants had dyslipidemia at the time of enrollment. Significant differences existed between people with and without dyslipidemia regarding BMI, TC, TG, HDL-C, and non-HDL-C levels (*P*< 0.001). A higher proportion of dyslipidemic subjects had diabetes compared to those without dyslipidemia (*P*< 0.001). Among all 676 participants with dyslipidemia at baseline, there were 23.8% who had high levels of TC, 50% who had increased TG, 46% who had low levels of HDL-C, and 17.3% who had increased non-HDL-C. During the follow-up, 216 subjects in the dyslipidemia group and 421 in the non-dyslipidemia group developed hypertension. The incidence rate of hypertension in the dyslipidemic population was 378.21 per 10000 person-years, while 336.89 per 10000 person-years in the non-dyslipidemic population (**Supplementary Table 1**).

**Table 1:** Baseline characteristics of the study population by dyslipidemia status.

Characteristic	Total(N=2116)	Dyslipidemia		*Z/χ^2^*	*P*
		No (n=1440)	Yes (n=676)		
Age (years)	57.65 (50.83, 64.45)	57.73 (51.08, 64.04)	57.32 (50.81, 64.78)	-0.297	0.767
Age<55 years	856 (40.45)	567 (39.38)	289 (42.75)	2.127	0.145
Male (%)	853 (40.31)	589 (40.90)	264 (39.05)	0.685	0.408
Smoker (%)	525 (24.81)	373 (25.90)	152 (22.49)	3.073	0.080
Drinker (%)	468 (22.12)	328 (22.78)	140 (20.71)	1.282	0.258
Hypertension family history (%)	408 (19.28)	284 (19.72)	124 (18.34)	0.497	0.481
Diabetes (%)	193 (9.12)	105 (7.29)	88 (13.02)	18.479	<0.001
BMI (kg/m^2^)	23.42 (21.49, 25.85)	23.03 (21.11, 25.46)	24.38 (22.42, 26.49)	7.824	<0.001
SBP (mmHg)	127 (119, 133)	126 (119, 133)	127 (120, 133)	1.479	0.139
DBP (mmHg)	80 (76, 83)	80 (76, 83)	80 (77, 84)	2.508	0.012
FPG (mmol/L)	5.22 (4.78, 5.67)	5.22 (4.78, 5.64)	5.22 (4.79, 5.84)	1.566	0.117
TC (mg/dl)	183.98 (161.00, 207.34)	181.66 (161.78, 201.16)	190.93 (157.34, 239.19)	6.257	<0.001
TG (mg/dl)	105.31 (73.45, 161.95)	90.27 (65.93, 124.78)	200.00 (118.14, 271.24)	23.784	<0.001
LDL-C (mg/dl)	100.00 (84.17, 117.76)	100.00 (86.10, 115.83)	100.00 (78.38, 127.80)	0.445	0.656
HDL-C (mg/dl)	51.35 (44.02, 59.85)	53.67 (47.10, 61.00)	42.47 (36.68, 55.60)	-16.344	<0.001
Non-HDL-C (mg/dl)	131.08 (110.04, 153.67)	126.06 (106.56, 145.17)	145.14 (118.53, 178.38)	12.437	<0.001
TC group(%)				371.239	<0.001
Normal (<200)	1434 (67.77)	1058 (73.47)	376 (55.62)		
Marginal increase (200-240)	521 (24.62)	382 (26.53)	139 (20.56)		
Increase (>240)	161 (7.61)	0 (0)	161 (23.82)		
TG group(%)				877.083	<0.001
Normal (<150)	1504 (71.08)	1250 (86.82)	254 (37.57)		
Marginal increase (150-200)	274 (12.95)	190 (13.19)	84 (12.43)		
Increase (>200)	338 (15.97)	0 (0)	338 (50.00)		
LDL-C group(%)				144.594	<0.001
Normal (<130)	1837 (86.81)	1315 (91.32)	522 (77.22)		
Marginal increase (130-160)	223 (10.54)	125 (8.68)	98 (14.50)		
Increase (>160)	56 (2.65)	0 (0)	56 (8.28)		
HDL-C group(%)				780.561	<0.001
Normal (40-60)	1280 (60.49)	1039 (72.15)	241 (35.65)		
Low (<40)	311 (14.70)	0 (0)	311 (46.01)		
High (>60)	525 (24.81)	401 (27.85)	124 (18.34)		
Non-HDL-C group(%)				338.293	<0.001
Normal (<160)	1706 (80.62)	1294 (89.86)	412 (60.95)		
Marginal increase (160-190)	292 (13.80)	145 (10.07)	147 (21.75)		
Increase (>190)	118 (5.58)	1 (0.07)	117 (17.31)		

Values are presented as median (interquartile range) or n (%).

BMI, body mass index; DBP, diastolic blood pressure; FPG, fasting plasma glucose; HDL-C, high-density lipoprotein cholesterol; LDL-C, low-density lipoprotein cholesterol; Non-HDL-C, non-high-density lipoprotein cholesterol; SBP, systolic blood pressure; TC, total cholesterol; TG, triglycerides.

### Association between lipid indices and hypertension in the whole population

As of July 27, 2020, the median follow-up period was 10.75 years and 637 participants developed hypertension with an incidence density of 349.85 per 10000 person-years. **Table 2** shows the crude and adjusted results of Cox regression analyses in the whole study population. The univariable analysis suggested that the TC level between 200 and 240 mg/dl was significantly associated with an elevated risk of incident hypertension (unadjusted HR [95%CI]: 1.256[1.051-1.500]). Yet, when we added covariates (age, gender, smoking, drinking, hypertension family history, and diabetes) to the model, none of the lipid indices were significantly associated with new-onset hypertension.

**Table 2:** Association between lipid indices and hypertension incidence during follow-up in the whole study population.

Lipid Indices	Group	Unadjusted		Adjusted	
		HR (95%CI)	*P*	HR (95%CI)	*P*
TC	<200 mg/dl	Reference	–	Reference	–
	200-240 mg/dl	1.256 (1.051-1.500)	0.012	1.182 (0.986-1.416)	0.070
	>240 mg/dl	1.234 (0.927-1.643)	0.149	1.043 (0.778-1.398)	0.779
TG	<150 mg/dl	Reference	–	Reference	–
	150-200 mg/dl	1.225 (0.979-1.533)	0.076	1.153 (0.918-1.448)	0.221
	>200 mg/dl	1.163 (0.944-1.433)	0.156	1.129 (0.912-1.398)	0.265
LDL-C	<130 mg/dl	Reference	–	Reference	–
	130-160 mg/dl	1.177 (0.923-1.501)	0.188	1.047 (0.819-1.338)	0.714
	>160 mg/dl	1.291 (0.817-2.039)	0.274	1.249 (0.790-1.975)	0.341
HDL-C	40-60 mg/dl	Reference	–	Reference	–
	<40 mg/dl	1.215 (0.978-1.511)	0.079	1.145 (0.920-1.426)	0.224
	>60 mg/dl	1.116 (0.929-1.341)	0.240	1.095 (0.909-1.319)	0.340
Non-HDL-C	<160 mg/dl	Reference	–	Reference	–
	160-190 mg/dl	1.191 (0.958-1.482)	0.116	1.107 (0.886-1.382)	0.371
	>190 mg/dl	1.163 (0.839-1.611)	0.364	0.960 (0.688-1.340)	0.812

Covariates in the adjusted model were age, gender, smoking, drinking, hypertension family history, and diabetes.

TC, total cholesterol; TG, triglycerides; LDL-C, low-density lipoprotein cholesterol; HDL-C, high-density lipoprotein cholesterol; Non-HDL-C, non-high-density lipoprotein cholesterol; HR, hazard ratio; CI, confidence interval.

### Age stratified analysis of association between lipid indices and hypertension

The bivariate correlation between lipid indices and hypertension stratified by baseline age is presented in **Supplementary Table 2**. In the population aged< 55 years, TC, TG, LDL-C, and non-HDL-C were significantly related to hypertension incidence (*P*< 0.05). In contrast, among those older than 55 years, none of the lipid indices had a significant relationship with hypertension (*P* > 0.05). In **Table 3**, Cox regression analyses with adjustment for covariates indicated that, within the younger age group, marginally elevated TG (150-200 mg/dl) and LDL-C (130-160 mg/dl) levels, elevated TC (>240 mg/dl) and non-HDL-C (>190 mg/dl) levels were significantly associated with the increased risk of hypertension compared to normal lipid levels. Adjusted HRs (95%CIs) were 1.659 (1.121-2.457), 1.691 (1.094-2.612), 2.744 (1.716-4.386), and 2.156 (1.281-3.628), respectively. For those aged ≥ 55 years, no significant association was found between abnormal lipid levels and the risk of developing hypertension (*P* > 0.05).

**Table 3:** Multivariable association between lipid indices and the risk of hypertension by age groups.

**Lipid Indices**	**Group**	**Age < 55 years**			**Age ≥ 55 years**		
		**β Estimate**	**HR (95%CI)**	***P***	**β Estimate**	**HR (95%CI)**	***P***
TC	<200 mg/dl	–	Reference	–	–	Reference	–
	200-240 mg/dl	0.262	1.299 (0.919-1.838)	0.139	0.105	1.110 (0.896-1.376)	0.338
	>240 mg/dl	1.009	2.744 (1.716-4.386)	<0.001	-0.279	0.757 (0.522-1.097)	0.141
TG	<150 mg/dl	–	Reference	–	–	Reference	–
	150-200 mg/dl	0.506	1.659 (1.121-2.457)	0.011	0.052	1.053 (0.795-1.394)	0.718
	>200 mg/dl	0.273	1.314 (0.910-1.898)	0.145	0.180	1.197 (0.924-1.550)	0.173
LDL-C	<130 mg/dl	–	Reference	–	–	Reference	–
	130-160 mg/dl	0.525	1.691 (1.094-2.612)	0.018	-0.090	0.914 (0.680-1.229)	0.552
	>160 mg/dl	0.661	1.936 (0.940-3.988)	0.073	-0.007	0.993 (0.545-1.809)	0.982
HDL-C	40-60 mg/dl	–	Reference	–	–	Reference	–
	<40 mg/dl	0.122	1.130 (0.765-1.669)	0.538	0.216	1.241 (0.952-1.617)	0.111
	>60 mg/dl	0.014	1.014 (0.703-1.462)	0.941	-0.001	0.999 (0.805-1.238)	0.990
Non-HDL-C	<160 mg/dl	–	Reference	–	–	Reference	–
	160-190 mg/dl	0.276	1.318 (0.864-2.010)	0.200	0.057	1.058 (0.814-1.376)	0.673
	>190 mg/dl	0.768	2.156 (1.281-3.628)	0.004	-0.262	0.769 (0.501-1.181)	0.231

Adjusted covariates were age, gender, smoking, drinking, hypertension family history, and diabetes.

TC, total cholesterol; TG, triglycerides; LDL-C, low-density lipoprotein cholesterol; HDL-C, high-density lipoprotein cholesterol; Non-HDL-C, non-high-density lipoprotein cholesterol; HR, hazard ratio; CI, confidence interval.

### Effects of lipid risk score on the risk of hypertension

**Figure 1:**
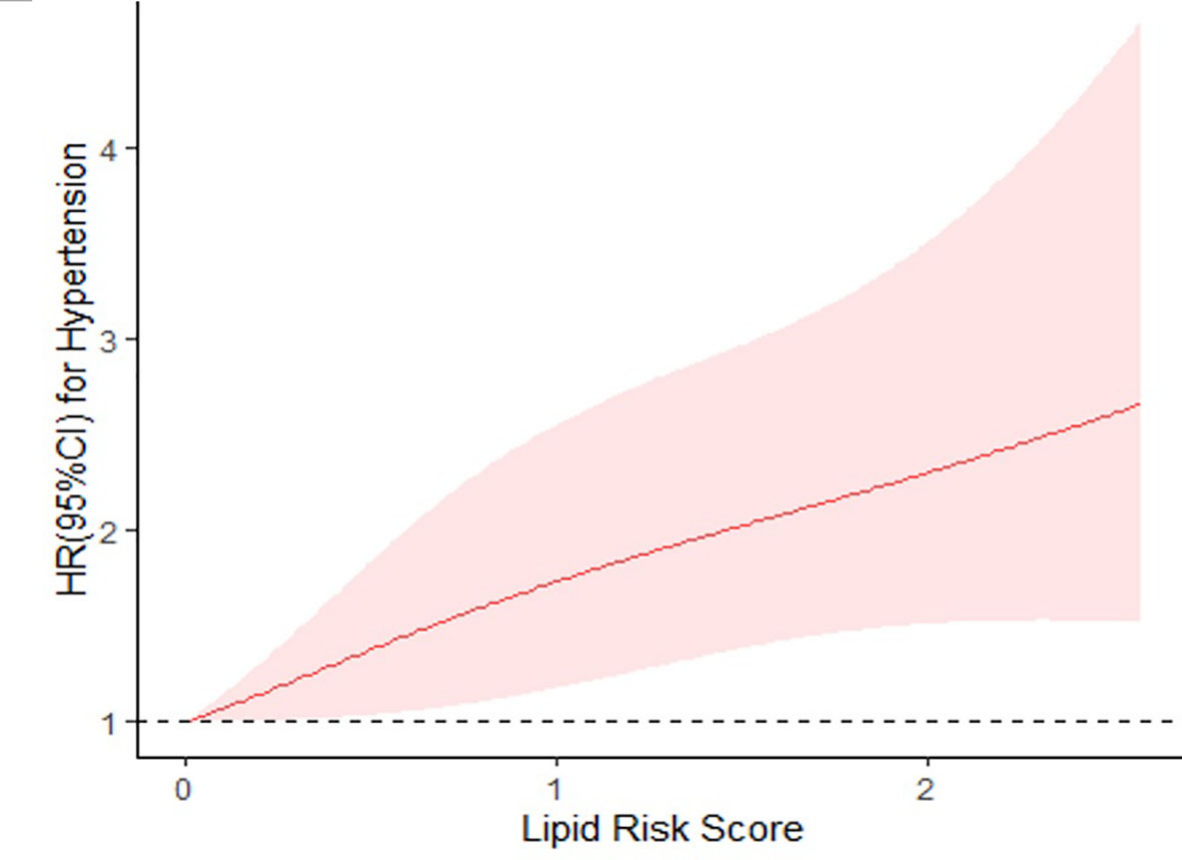
Restricted cubic spline regression of lipid risk score and hypertension. HRs are indicated by solid lines and 95% CIs by shaded areas. HR, hazard ratio; CI, confidence interval. Adjusted for age, gender, smoking, drinking, hypertension family history, diabetes at baseline, body mass index. *P* for overall association< 0.01; *P* for non-linearity = 0.4107; *P* for linear trend< 0.001.

In **Figure 1**, we used the restricted cubic spline to continuously model and visualize the relationship of lipid risk score with hypertension in the population under 55 years. The curve displayed a statistically significant linear trend (*P<* 0.001), which indicated that the lipid risk score had a positive linear correlation with hypertension. The hazard of developing hypertension tended to increase as the lipid risk score was getting higher.

Based on the result of restricted cubic spline analysis, we divided the lipid risk score (range: 0-2.81) into four categories with equal intervals, generating four risk groups for hypertension (**Supplementary Table 3**). During the follow-up, the incidence rate of hypertension increased from the first group to the fourth group, and the fourth group with the highest lipid risk score had the highest incidence rate (**Supplementary Table 4**). Results of multivariable Cox regression analyses showed that, in the< 55 years group, with every increase of 0.94 in lipid risk score, the risk of hypertension increased by 37% (adjusted HR [95%CI]: 1.369[1.164-1.610]). Compared with individuals who had normal lipid levels (lipid risk score = 0), those with lipid risk scores ranging from 0.94 to 1.87 had 2.19 times the risk of developing hypertension (HR [95%CI]: 2.192[1.278-3.760]), and those with lipid risk score > 1.87 had a 2.39-fold risk for hypertension (HR [95%CI]: 2.385[1.374-4.140]). **(** **Table 4** **)**

**Table 4:** Multivariable analyses of lipid risk score and incident hypertension in population< 55 years.

Variables	Class	β Estimate	HR (95%CI)	*P*
Lipid risk score	0	–	Reference	–
	0.01~	0.305	1.357 (0.984-1.871)	0.063
	0.94~	0.785	2.192 (1.278-3.760)	0.004
	1.87~	0.869	2.385 (1.374-4.140)	0.002
	Trend	0.314	1.369 (1.164-1.610)	*P*_trend_<0.001
Age		0.061	1.063 (1.023-1.104)	0.002
Gender	Female vs Male	0.234	1.264 (0.766-2.085)	0.359
Smoking	Yes vs No	0.379	1.461 (0.862-2.476)	0.159
Drinking	Yes vs No	0.015	1.015 (0.647-1.595)	0.947
Hypertension family history	Yes vs No	0.339	1.404 (1.020-1.932)	0.037
Diabetes	Yes vs No	0.124	1.132 (0.662-1.937)	0.651
BMI		0.054	1.055 (1.008-1.105)	0.022
SBP		0.013	1.014 (0.998-1.030)	0.096
DBP		0.008	1.009 (0.981-1.037)	0.551

HR, hazard ratio; CI, confidence interval; BMI, body mass index; SBP, systolic blood pressure; DBP, diastolic blood pressure.

We also evaluated the predictive effect of the lipid risk score for hypertension by comparing the Cox regression model with commonly included traditional risk factors (age, gender, smoking, drinking, hypertension family history, diabetes, BMI, SBP, DBP) and the model with lipid risk score added. C-statistic for the traditional model was 0.624 and C-statistic for the combination model was 0.651. The likelihood ratio test for these two models manifested that the lipid risk score had a significantly independent effect when added to the traditional risk model ( *χ* ^2^ =13.503, *P*< 0.001), and the model with lipid risk score plus traditional factors was statistically fitted.

### BMI stratification analysis of the association between lipid risk score and hypertension

**Table 5** lists the Cox regression analysis results of the continuous lipid risk score and hypertension stratified by the baseline BMI level of participants. Analyses were adjusted for age, gender, smoking, drinking, family history of hypertension as well as diabetes, and were conducted in the population younger than 55 years. There were significant associations between the lipid risk score and incident hypertension in both the normal-weight group (HR [95%CI]: 1.690[1.228-2.327]) and the overweight/obese group (HR [95%CI]: 1.375[1.064-1.778]). The association between lipid risk score and hypertension was slightly stronger in the normal-weight group than that in the overweight/obese group.

**Table 5:** Association between lipid risk score and hypertension by BMI groups.

Variables	Normal weight (n=462)		Overweight/Obese (n=394)	
	HR (95%CI)	*P*	HR (95%CI)	*P*
Lipid risk score	1.690 (1.228-2.327)	0.001	1.375 (1.064-1.778)	0.015
Age	1.062 (1.003-1.124)	0.040	1.067 (1.014-1.122)	0.012
Gender	1.268 (0.565-2.847)	0.564	1.191 (0.632-2.242)	0.589
Smoking	1.720 (0.745-3.971)	0.204	1.256 (0.625-2.525)	0.522
Drinking	1.183 (0.608-2.304)	0.620	0.854 (0.462-1.579)	0.614
Hypertension family history	1.192 (0.739-1.921)	0.472	1.594 (1.041-2.442)	0.032
Diabetes	0.960 (0.384-2.404)	0.931	1.149 (0.594-2.222)	0.680

HR, hazard ratio; CI, confidence interval.

## Discussion

In the present study, we prospectively investigated the relationship between integrated lipid indices and the risk of incident hypertension stratified by age. Our results demonstrated that the lipid risk score generated by combining TC, TG, LDL-C, and non-HDL-C had a positive linear correlation with hypertension. In the population younger than 55 years, those with higher lipid risk scores had a significantly increased risk of developing hypertension in the future. Of note, the comprehensive lipid score independent of traditional risk factors had a significantly predictive effect on hypertension incidence.

The development of hypertension is determined by a complicated synergy of multiple risk factors rather than a specific independent cause. Thus, when we intend to focus on the effect of a certain risk factor, the stratification analysis is an effective measure to reduce the influence of confounders in the observational study where there is no control group. Besides, evaluating the risk of hypertension in stratified groups based on its risk factors would lead to more targeted interventions and more rational allocation of limited medical resources than the general management. In this study, we stratified the study population by baseline age and analyzed the effects of lipid indices on the risk of developing hypertension in different age groups. Our current results suggested that significant associations existed between increased TC, TG, LDL-C, and non-HDL-C levels and the elevated risk of hypertension in the population under 55 years, while no significant association was detected between any lipid indices and hypertension for those older than 55 years. This is similar to a previous Brisighella Heart Study which found significant associations between LDL-C and blood pressure in individuals< 52 years and found no association in older individuals (25). And these significant associations in our study of lipid indices with hypertension in age-stratified analyses were not found in the whole population analysis when adjusted for confounders. Aging is an inevitable biological process and an important risk factor in most health disorders including hypertension and dyslipidemia. Large and long follow-up studies showed that the prevalence of hypertension increased dramatically with aging. Older adults account for the majority of hypertension-related morbidity and mortality (26, 27). Vascular dysfunction and arterial stiffness due to the aging of vessels, as well as chronic inflammation and increased cellular oxidative stress due to the weakening of physiological functions, play dominant roles in the development of hypertension among the elderly (26), which attenuates the effect of dyslipidemia. This might contribute to the reason why the relationship between lipid indices and hypertension was masked among people older than 55 years.

Higher BMI or excessive weight gain is also a well-known risk factor for both hypertension and dyslipidemia (28). So, to eliminate the moderating effects of overweight or obesity, we also conducted the stratification analysis by BMI levels and evaluated the independent effects of integrated lipid indices on hypertension. Our results detected significant associations between the lipid risk score and incident hypertension in both the normal-weight group and the high-BMI group, which implied that this association of lipid risk score with hypertension was not modified by overweight or obesity.

Hypertension and dyslipidemia are both independent and modifiable risk factors of great importance for atherosclerotic cardiovascular diseases (29–32). Abnormal changes in blood lipid levels are usually accompanied by elevation of blood pressure (33, 34). Previous cohort studies consistently identified the predictive effects of traditional or derived lipid indices on the future development of hypertension (16–18, 35). A Japanese study conducted in a working-age male population with a 4-year follow-up reported that people in the higher quintile of serum TC, LDL-C, and non-HDL-C had a higher risk for hypertension in multi-adjusted analyses (20). Another Chinese community-based non-hypertensive cohort study found that the risk of developing hypertension during follow-up increased with the increment of TG (22). The situations of TC, LDL-C, and non-HDL-C similarly occurred in our analyses. However, the result of the association between TG and hypertension in this study was equivocal, which was also reported in the aforementioned Japanese study. Nonetheless, our study was not aimed at finding a definite explanation for the reason why marginal increased TG was significantly associated with hypertension while the higher level of TG had no significant association. Besides, the heterogeneity test for the TG categories of marginal increase and increase did not detect heterogeneity between the HRs and 95% CIs of these two groups (Q = 0.7207, *P* = 0.3959), which indicated the rationality of using the averaged β estimate for the weight of TG in the calculation of lipid risk score. Another finding of our study worth mentioning was the correlation between LDL-C and hypertension. Though the P-value for the category of LDL-C >160 mg/dl was not significant, the HR and β estimate still showed a potentially stronger association of hypertension with the increased level than the marginally increased level (*P_trend_* = 0.005). This situation was probably due to the very few subjects in the group of LDL-C higher than 160 mg/dl with only 8 out of 23 individuals developing hypertension during follow-up.

Pathophysiological mechanisms contributing to the relationship between abnormal lipid profiles and elevated risk of hypertension mainly involve the dysfunction of vascular endothelium, RAAS activation, and insulin resistance. Endothelial dysfunction, manifested as deteriorated nitric oxide (NO) cascade (36), has always been believed in many studies to be a critical part in the correlation between lipid abnormalities and hypertension. Studies have shown that plasma cholesterols significantly correlate with endothelial NO synthase (eNOS) and NO activity. Increased LDL oxidation in hypercholesterolemic conditions could decrease the synthesis of eNOS, while HDL particles could increase NO production by stimulating eNOS activity (37, 38). Substantial evidence supports the direct contribution of endothelial dysfunction to the pathogenesis of hypertension *via* the imbalance between vasodilatory and vasocontrictory substances (36, 39, 40). The consequent increase of systemic vascular resistance leads directly to the elevation of blood pressure. Moreover, there is growing evidence for the involvement of the renin-angiotensin system in the correlation between hypercholesterolemia and hypertension. Hypercholesterolemia fosters the RAAS activity and the synthesis of angiotensin and endothelin (37, 41). Insulin resistance is another possible mechanism connecting dyslipidemia and hypertension, which usually exists in obese or diabetic patients with high plasma TG levels or impaired glucose tolerance (42, 43). Some researchers believed that secondary hyperinsulinemia induced by insulin resistance is responsible for the increment in blood pressure (44). In addition to the three aforementioned mechanisms, renal microvascular injury induced by lipid abnormalities or dyslipoproteinemia also make a difference in the development of hypertension (45–47). Some researchers investigated the genetic association and metabolomic patterns between hyperlipidemia and hypertension which undeniably are promising research directions (14, 48).

Based on the intricate mechanisms of the relationship between dyslipidemia and hypertension, many kinds of cholesterols and triglycerides participate in the pathogenesis. Though previous research indicated that TC, TG, LDL-C, HDL-C, or non-HDL-C, individually, has some relation to the risk of hypertension, it is necessary to investigate the comprehensive effects of lipid profiles and hypertension. Therefore, our research evaluated the combined effects of multiple lipid indices on the risk of incident hypertension. And we found that with every increase of 0.94 in lipid risk score, the risk of hypertension increased by 37% in the population younger than 55 years. The lipid risk score integrated several lipid indices weighted by effect sizes, which will help depict the lipid characteristic for each individual more accurately.

For multi-factorial disorders like hypertension, scholars around the world never cease to pursue the optimal prediction model to precisely assess the risk of disease for individuals. All kinds of risk prediction models have been developed from equations to risk scores, from Western populations to Asian populations. However, based on the systematic review, only a few models included lipid indices (23). So, our study tried to add the integrated lipid score to the traditional model of hypertension, and we found a small increment in the C-statistics. Our results accord with previous studies which also detected better discrimination with the addition of lipid variables (49, 50). Thus, we have reason to infer that it is very likely that adding lipid-related factors to the traditional risk model could slightly improve the performance of the prediction model for hypertension in certain populations.

Though this study has notable strengths, some limitations are worth mentioning. First, the sample size of this study was still relatively small and all subjects were recruited from Jiangsu Province in China, which is a lack of representativeness and external validity. Thus, studies with larger sample sizes and different populations are warranted to validate these findings. Second, since the lipid data for analyses were collected at baseline, we were not able to evaluate the effect of lipid level changes on the development of hypertension. Third, a potential confounding bias is also noticeable. The possible use of lipid-lowering medications during the follow-up period might distort the relationship between dyslipidemia and new-onset hypertension. Fourth, the median LDL-C levels at baseline in groups with or without dyslipidemia were the same due to the low proportion of dyslipidemia subjects having LDL-C levels higher than normal. In this situation, the effect of elevated LDL-C could not have a clear manifestation in the correlation between dyslipidemia and incident hypertension. Moreover, the selected cut-off values and categorization of lipid index levels in this study have to some extent masked the effect of lipid levels above-median yet currently classified in the normal group on the risk of hypertension. Additionally, restricted by the sample size of our study, HDL-C was not found to have significant association with incident hypertension in any population. So, we did not include HDL-C in the calculation of lipid risk score in this study, which might potentially make our lipid risk score less effective and less comprehensive. Finally, lipid indices included in this study are still limited, so further analyses of the correlation between hypertension and nonconventional or derived lipid indices such as TC/HDL-C are necessary.

In conclusion, the current results of this prospective study provide convincing evidence for the causal relationship between dyslipidemia and hypertension, as well as the combined effects of multiple lipid indices on hypertension. Our findings may also contribute to the proposition that the risk score with integrated lipid indices could serve as an independent risk factor of developing hypertension. Therefore, the lipid risk score combined with traditional risk factors could delineate a more comprehensive and more accurate risk profile for hypertension, which may aid in optimizing the risk prediction model and identifying the high-risk individuals of hypertension. In addition, the comprehensive risk assessment will help improve individuals’ prophylactic awareness and compliance, as well as facilitate personalized intervention strategies. This study also highlights the importance of integrated management of dyslipidemia and hypertension. Comprehensive lipid controls should be paid more attention in the prevention of hypertension. Nevertheless, future clinical applications of the finding still need corroboration and adjustment in more and larger studies. Moreover, the extent to which treatment of dyslipidemia reduces the risk of developing hypertension is also worth exploring.

## Data availability statement

The raw data supporting the conclusions of this article will be made available by the authors, without undue reservation.

## Ethics statement

The studies involving human participants were reviewed and approved by Nanjing Medical University (#200803307). The patients/participants provided their written informed consent to participate in this study. The study was conducted in accordance with the Declaration of Helsinki, and approved by the Ethics Committee of Nanjing Medical University (#200803307), Nanjing, China. Written informed consent has been obtained from all participants to publish this paper.

## Author contributions

Conceptualization, CS and SY; methodology, CS; software, HX; formal analysis, HX; investigation, QZ, JS, PW, XZ, YC, JD, CC, JM, LW, YY; resources, CS and SY; data curation, JD; writing—original draft preparation, HX; writing—review and editing, CS; visualization, HX; supervision, CS; project administration, CS and SY; funding acquisition, CS. All authors contributed to the article and approved the submitted version.

## Funding

This research was funded by the National Key Research and Development Program of China (Grant No. 2018YFC2000703), Research Unit of Prospective Cohort of Cardiovascular Diseases and Cancers of Chinese Academy of Medical Sciences (2019RU038). National Natural Science Foundation of China (Grant No. 81872686, No.82173611, and No. 81573232), the Priority Academic Program for the Development of Jiangsu Higher Education Institutions (Public Health and Preventive Medicine), and the Flagship Major Development of Jiangsu Higher Education Institutions.

## Acknowledgments

The authors would like to express gratitude to all participants in this study for providing valuable data and information, and to the clinical staff at the People’s Hospital of Yixing City for their support.

## Conflict of interest

The authors declare that the research was conducted in the absence of any commercial or financial relationships that could be construed as a potential conflict of interest.

## Publisher’s note

All claims expressed in this article are solely those of the authors and do not necessarily represent those of their affiliated organizations, or those of the publisher, the editors and the reviewers. Any product that may be evaluated in this article, or claim that may be made by its manufacturer, is not guaranteed or endorsed by the publisher.
