# Supplementary material for: The relationship between lipid risk score and new-onset hypertension in a prospective cohort study
Source: Front Endocrinol (Lausanne). 2022 Sep 28;13:916951. doi: 10.3389/fendo.2022.916951 (PMC9555054; doi:10.3389/fendo.2022.916951)
Supplement: Supplementary file 1 [file DataSheet_1.docx]

**Supplementary Table 1.**

Hypertension incidence in population with and without dyslipidemia by age groups

| **Dyslipidemia** | **Total** | | |  | **Age < 55 years** | | |  | **Age ≥ 55 years** | | |
| --- | --- | --- | --- | --- | --- | --- | --- | --- | --- | --- | --- |
|  | **Hypertension Cases** | **Person Years** | **Incidence Density (per 10000 person years)** |  | **Hypertension Cases** | **Person Years** | **Incidence Density (per 10000 person years)** |  | **Hypertension Cases** | **Person Years** | **Incidence Density (per 10000 person years)** |
| All | 637 | 18207.9 | 349.85 |  | 186 | 8097.6 | 229.70 |  | 451 | 10110.3 | 446.08 |
| Yes | 216 (33.9) | 5711.0 | 378.21 |  | 72 | 2641.2 | 272.61 |  | 144 | 3069.9 | 469.07 |
| No | 421 (66.1) | 12496.8 | 336.89 |  | 114 | 5456.4 | 208.93 |  | 307 | 7040.4 | 436.05 |

**Supplementary Table 2.**

Univariate analyses of lipid indices and hypertension incidence by age groups

| **Lipid Indices** | **Group** | **Age ＜ 55 years** | | | |  | **Age ≥ 55 years** | | | |
| --- | --- | --- | --- | --- | --- | --- | --- | --- | --- | --- |
|  |  | **Hypertension Cases** | **Person Years** | **Incidence Density (per 10000 person years)** | ***P*** |  | **Hypertension Cases** | **Person Years** | **Incidence Density (per 10000 person years)** | ***P*** |
| TC | <200 mg/dl | 119 | 6058.83 | 196.41 | <0.001 |  | 292 | 6483.55 | 450.37 | 0.237 |
|  | 200-240 mg/dl | 46 | 1654.50 | 278.03 |  |  | 127 | 2679.59 | 473.95 |  |
|  | >240 mg/dl | 21 | 384.26 | 546.50 |  |  | 32 | 947.15 | 337.86 |  |
| TG | <150 mg/dl | 114 | 5682.67 | 200.61 | 0.027 |  | 319 | 7357.03 | 433.60 | 0.498 |
|  | 150-200 mg/dl | 33 | 979.51 | 336.90 |  |  | 60 | 1314.45 | 456.47 |  |
|  | >200 mg/dl | 39 | 1435.41 | 271.70 |  |  | 72 | 1438.81 | 500.41 |  |
| LDL-C | <130 mg/dl | 154 | 7259.30 | 212.14 | 0.016 |  | 390 | 8652.44 | 450.74 | 0.786 |
|  | 130-160 mg/dl | 24 | 647.04 | 370.92 |  |  | 50 | 1199.37 | 416.88 |  |
|  | >160 mg/dl | 8 | 191.27 | 418.26 |  |  | 11 | 258.48 | 425.57 |  |
| HDL-C | 40-60 mg/dl | 113 | 5163.40 | 218.85 | 0.756 |  | 255 | 6023.11 | 423.37 | 0.144 |
|  | <40 mg/dl | 33 | 1339.52 | 246.36 |  |  | 71 | 1271.82 | 558.26 |  |
|  | >60 mg/dl | 40 | 1594.68 | 250.83 |  |  | 125 | 2815.36 | 443.99 |  |
| Non-HDL-C | <160 mg/dl | 144 | 6835.84 | 210.65 | 0.009 |  | 358 | 7927.71 | 451.58 | 0.533 |
|  | 160-190 mg/dl | 26 | 923.25 | 281.61 |  |  | 70 | 1519.48 | 460.68 |  |
|  | >190 mg/dl | 16 | 338.52 | 472.65 |  |  | 23 | 663.10 | 346.86 |  |

Abbreviations: TC, total cholesterol; TG, triglycerides; LDL-C, low-density lipoprotein cholesterol; HDL-C, high-density lipoprotein cholesterol; Non-HDL-C, non-high-density lipoprotein cholesterol.

**Supplementary Table 3.**

Baseline characteristics and lipid risk score of four lipid risk groups in the population under 55 years

| **Characteristic** | **Total** | **Lipid Risk Groups *** | | | |
| --- | --- | --- | --- | --- | --- |
|  |  | **1 [0.00-0.00]** | **2 [0.01-0.94]** | **3 [0.94-1.87]** | **4 [1.87-2.81]** |
| N (%) | 856 (100.00) | 445 (51.99) | 323 (37.73) | 48 (5.61) | 40 (4.67) |
| Lipid Risk Score | 0.00 (0.00, 0.37) | 0.00 (0.00, 0.00) | 0.37 (0.37, 0.54) | 1.16 (1.06, 1.44) | 2.37 (2.15, 2.68) |
| Dyslipidemia (%) | 289 (33.76) | 66 (14.83) | 156 (48.30) | 27 (56.25) | 40 (100.0) |
| Male (%) | 294 (34.35) | 141 (31.69) | 120 (37.15) | 21 (43.75) | 12 (30.00) |
| Smoker (%) | 197 (23.01) | 109 (24.49) | 71 (21.98) | 12 (25.00) | 5 (12.50) |
| Drinker (%) | 191 (22.31) | 94 (21.12) | 77 (23.84) | 13 (27.08) | 7 (17.50) |
| Hypertension family history (%) | 215 (25.12) | 105 (23.60) | 94 (29.1) | 10 (20.83) | 6 (15.00) |
| Diabetes (%) | 62 (7.24) | 24 (5.39) | 29 (8.98) | 2 (4.17) | 7 (17.50) |
| Age (years) | 48.44 (45.83, 52.69) | 47.75 (45.76, 52.00) | 49.41 (46.24, 52.73) | 51.80 (46.75, 53.03) | 50.81 (46.20, 53.37) |
| BMI (kg/m^2^) | 23.63 (21.83, 25.91) | 23.15 (21.50, 25.39) | 24.09 (22.27, 26.22) | 25.06 (22.63, 27.52) | 24.87 (22.02, 27.83) |
| SBP (mmHg) | 125 (118, 132) | 125 (119, 132) | 125 (118, 131) | 125 (115, 132.25) | 129 (119, 134.50) |
| DBP (mmHg) | 80 (77, 83) | 80 (76, 83) | 80 (77, 83) | 79 (73.5, 84.5) | 80 (78, 82.75) |
| TC (mg/dl) | 179.54 (155.98, 201.54) | 164.86 (148.65, 180.31) | 194.59 (172.20, 209.27) | 232.63 (220.85, 238.8) | 264.67 (247.1285.52) |
| TG (mg/dl) | 106.64 (76.11, 169.47) | 84.96 (65.49, 104.42) | 172.57 (122.12, 238.94) | 139.38 (88.05, 187.17) | 249.56 (196.02, 348.23) |
| LDL-C (mg/dl) | 98.84 (83.01, 116.80) | 93.44 (79.92, 105.79) | 102.70 (84.56, 120.46) | 135.91 (130.50, 145.95) | 144.79 (122.20, 157.34) |
| HDL-C (mg/dl) | 50.19 (43.24, 57.92) | 50.19 (44.02, 57.14) | 49.03 (41.31, 57.92) | 54.63 (48.07, 63.13) | 53.86 (44.40, 62.36) |
| Non-HDL-C (mg/dl) | 127.41 (107.72, 148.26) | 114.67 (96.14, 127.03) | 143.24 (123.17, 155.60) | 172.97 (167.37, 181.66) | 205.79 (197.30, 229.34) |
| TC (%) |  |  |  |  |  |
| Normal (<200) | 628 (73.36) | 445(100.0) | 180 (55.73) | 3 (6.25) | 0 (0) |
| Marginal increase (200-240) | 179 (20.91) | 0 (0) | 143 (44.27) | 35 (72.92) | 1 (2.5) |
| Increase (>240) | 49 (5.72) | 0 (0) | 0 (0) | 10 (20.83) | 39 (97.5) |
| TG (%) |  |  |  |  |  |
| Normal (<150) | 590 (68.93) | 445 (100.0) | 109 (33.75) | 29 (60.42) | 7 (17.5) |
| Marginal increase (150-200) | 111 (12.97) | 0 (0) | 100 (30.96) | 8 (16.67) | 3 (7.5) |
| Increase (>200) | 155 (18.11) | 0 (0) | 114 (35.29) | 11 (22.92) | 30 (75.0) |
| LDL-C (%) |  |  |  |  |  |
| Normal (<130) | 759 (88.67) | 445 (100.0) | 292 (90.4) | 10 (20.83) | 12 (30.0) |
| Marginal increase (130-160) | 74 (8.64) | 0 (0) | 24 (7.43) | 31 (64.58) | 19 (47.5) |
| Increase (>160) | 23 (2.69) | 0 (0) | 7 (2.17) | 7 (14.58) | 9 (22.5) |
| HDL-C (%) |  |  |  |  |  |
| Normal (40-60) | 540 (63.08) | 304 (68.31) | 186 (57.59) | 28 (58.33) | 22 (55) |
| Low (<40) | 144 (16.82) | 64 (14.38) | 69 (21.36) | 6 (12.5) | 5 (12.5) |
| High (>60) | 172 (20.09) | 77 (17.3) | 68 (21.05) | 14 (29.17) | 13 (32.5) |
| Non-HDL-C (%) |  |  |  |  |  |
| Normal (<160) | 715 (83.53) | 445 (100.0) | 263 (81.42) | 7 (14.58) | 0 (0) |
| Marginal increase (160-190) | 100 (11.68) | 0 (0) | 59 (18.27) | 37 (77.08) | 4 (10.0) |
| Increase (>190) | 41 (4.79) | 0 (0) | 1 (0.31) | 4 (8.33) | 36 (90.0) |

*: Lipid risk groups were four categories of the lipid risk score (range: 0-2.81) with equal intervals.

Values are presented as median (interquartile range) or n (%). Abbreviations: BMI, body mass index; DBP, diastolic blood pressure; HDL-C, high-density lipoprotein cholesterol; LDL-C, low-density lipoprotein cholesterol; Non-HDL-C, non-high-density lipoprotein cholesterol; SBP, systolic blood pressure; TC, total cholesterol; TG, triglycerides.

**Supplementary Table 4.**

Hypertension incidence in four groups of lipid risk score during follow-up

| **Groups of Lipid Risk Score** | **N** | **Hypertension Cases** | **Person Years** | **Incidence Density**  **(per 10000 person years)** |
| --- | --- | --- | --- | --- |
| 1 [0.00-0.00] | 445 | 76 | 4361.8 | 174.24 |
| 2 [0.01-0.94] | 323 | 77 | 3012.6 | 255.59 |
| 3 [0.94-1.87] | 48 | 17 | 392.1 | 433.52 |
| 4 [1.87-2.81] | 40 | 16 | 331.1 | 483.31 |

**Supplementary Table 5.**

Baseline features of the population by hypertension status during follow-up

| **Characteristic** | **Total**  (N=2116) | **Non-hypertension**  (n=1479) | **Hypertension**  (n=637) | ***P* value** |
| --- | --- | --- | --- | --- |
| Age (years) | 57.65 (50.83, 64.45) | 55.83 (49.00, 62.77) | 60.15 (54.00, 67.33) | <0.001 |
| Male (%) | 853 (40.3) | 575 (38.9) | 278 (43.6) | 0.040 |
| Smoker (%) | 525 (24.8) | 358 (24.2) | 167 (26.2) | 0.326 |
| Drinker (%) | 468 (22.1) | 308 (20.8) | 160 (25.1) | 0.029 |
| Hypertension family history (%) | 408 (19.3) | 287 (19.4) | 121 (19.0) | 0.827 |
| Diabetes (%) | 193 (9.1) | 128 (8.7) | 65 (10.2) | 0.256 |
| BMI (kg/m^2^) | 23.42 (21.49, 25.85) | 23.19 (21.37, 25.56) | 24.00 (21.76, 26.44) | <0.001 |
| SBP (mmHg) | 127.00 (119.00, 133.00) | 125.00 (117.50, 132.00) | 129.00 (120.00, 135.00) | <0.001 |
| DBP (mmHg) | 80.00 (76.00, 83.00) | 80.00 (75.00, 83.00) | 80.00 (78.00, 84.00) | <0.001 |
| FPG (mmol/L) | 5.22 (4.78, 5.67) | 5.21 (4.78, 5.64) | 5.24 (4.76, 5.78) | 0.281 |
| TC (mg/dl) | 183.98 (161.00, 207.34) | 183.40 (160.23, 205.79) | 186.10 (163.32, 210.04) | 0.090 |
| TG (mg/dl) | 105.31 (73.45, 161.95) | 101.77 (70.80, 159.29) | 112.39 (79.65, 169.03) | 0.004 |
| LDL-C (mg/dl) | 100.00 (84.17, 117.76) | 99.61 (83.40, 117.37) | 101.16 (84.56, 118.92) | 0.362 |
| HDL-C (mg/dl) | 51.35 (44.02, 59.85) | 51.35 (44.02, 59.85) | 51.35 (43.24, 60.62) | 0.834 |
| Non-HDL-C (mg/dl) | 131.08 (110.04, 153.67) | 130.12 (109.65, 151.74) | 134.36 (111.20, 157.14) | 0.046 |
| TC (%) |  |  |  | 0.110 |
| Normal (<200) | 1434 (67.8) | 1023 (69.2) | 411 (64.5) |  |
| Marginal increase (200-240) | 521 (24.6) | 348 (23.5) | 173 (27.2) |  |
| Increase (>240) | 161 (7.6) | 108 (7.3) | 53 (8.3) |  |
| TG (%) |  |  |  | 0.113 |
| Normal (<150) | 1504 (71.1) | 1071 (72.4) | 433 (68.0) |  |
| Marginal increase (150-200) | 274 (12.9) | 181 (12.2) | 93 (14.6) |  |
| Increase (>200) | 338 (16.0) | 227 (15.3) | 111 (17.4) |  |
| LDL-C (%) |  |  |  | 0.448 |
| Normal (<130) | 1837 (86.8) | 1293 (87.4) | 544 (85.4) |  |
| Marginal increase (130-160) | 223 (10.5) | 149 (10.1) | 74 (11.6) |  |
| Increase (>160) | 56 (2.6) | 37 (2.5) | 19 (3.0) |  |
| HDL-C (%) |  |  |  | 0.202 |
| Normal (40-60) | 1280 (60.5) | 912 (61.7) | 368 (57.8) |  |
| Low (<40) | 311 (14.7) | 207 (14.0) | 104 (16.3) |  |
| High (>60) | 525 (24.8) | 360 (24.3) | 165 (25.9) |  |
| Non-HDL-C (%) |  |  |  | 0.382 |
| Normal (<160) | 1706 (80.6) | 1204 (81.4) | 502 (78.8) |  |
| Marginal increase (160-190) | 292 (13.8) | 196 (13.3) | 96 (15.1) |  |
| Increase (>190) | 118 (5.6) | 79 (5.3) | 39 (6.1) |  |

Values are presented as median (interquartile range) or n (%).

Abbreviations: BMI, body mass index; DBP, diastolic blood pressure; FPG, fasting plasma glucose; HDL-C, high-density lipoprotein cholesterol; LDL-C, low-density lipoprotein cholesterol; Non-HDL-C, non-high-density lipoprotein cholesterol; SBP, systolic blood pressure; TC, total cholesterol; TG, triglycerides.
